# Automatic Extraction of Appendix from Ultrasonography with Self-Organizing Map and Shape-Brightness Pattern Learning

**DOI:** 10.1155/2016/5206268

**Published:** 2016-04-12

**Authors:** Kwang Baek Kim, Doo Heon Song, Hyun Jun Park

**Affiliations:** ^1^Department of Computer Engineering, Silla University, Busan 46958, Republic of Korea; ^2^Department of Computer Games, Yong-in Songdam College, Yongin 17145, Republic of Korea; ^3^Department of Computer Engineering, Pusan National University, Busan 46241, Republic of Korea

## Abstract

Accurate diagnosis of acute appendicitis is a difficult problem in practice especially when the patient is too young or women in pregnancy. In this paper, we propose a fully automatic appendix extractor from ultrasonography by applying a series of image processing algorithms and an unsupervised neural learning algorithm, self-organizing map. From the suggestions of clinical practitioners, we define four shape patterns of appendix and self-organizing map learns those patterns in pixel clustering phase. In the experiment designed to test the performance for those four frequently found shape patterns, our method is successful in 3 types (1 failure out of 45 cases) but leaves a question for one shape pattern (80% correct).

## 1. Introduction

Appendicitis, one of the most common surgical abdominal emergences, is an inflammation of the appendix that can be classified into early appendicitis, gangrenous appendicitis, gangrenous appendicitis, chronic appendicitis, and acute appendicitis according to its development stage [1]. Typically, the illness begins with vague midabdominal discomfort followed by nausea, anorexia, and indigestion and within several hours the pain migrates to the right lower quadrant [2]. Examination at this point shows localized tenderness to one-finger palpation and perhaps slight muscular guarding. Rebound or percussion tenderness (the latter provides the same information more humanely) may be elicited in the same area [3].

Often the site of maximum tenderness is located at McBurney's point, at which lies two-thirds along a line from the umbilicus to the anterior superior iliac spine [4]. However, there are various kinds of difficulties in the diagnosis of acute appendicitis especially for high false-positive diagnosis rate in women aged between 20 and 40 [5] and women in pregnancy because the nausea, vomiting, and abdominal pain of appendicitis can also be features of pregnancy and physical examination may not be reliable in them [6].

Recent studies advocate the use of medical imaging to reduce the rate of negative appendectomies [7]. Among available imaging modalities such as ultrasonography (US), computed tomography (CT), and magnetic resonance imaging (MRI), US may be especially useful where there are equivocal clinical signs or an indeterminate diagnostic score. In these situations, US may improve diagnostic accuracy by reducing the number of false negatives and therefore prevent unnecessary surgery [8]. US examination should be the first imaging test performed, particularly among the pediatric and young adult populations [9], who represent the main targets for appendicitis, and in pregnant patients.

Among known sonographic findings of acute appendicitis, a threshold of 6 mm diameter of the appendix under compression is the most accurate US finding for appendicitis [10]. Thus, the critical point, 6 mm of the diameter of the appendix, is a crucial factor in decision making for appendectomy. As a result, the measurement error of 1 mm near the critical point may lead doctors to a serious misdiagnosis [2].

While the reliability of US in diagnosing acute appendicitis is much improved to be matched with that of CT or MRI [6], current naked eye examination of the US has limitations in accurate measurement in cases of unclear delineation of the appendix with thick abdomen and in cases showing ill-defined borders of the appendix by surrounding tissues and its intrinsic operator subjectivity [2].

Thus, there are growing needs for an intelligent decision making tool for more accurate diagnosis by artificial intelligence technology and careful image processing and analyzing algorithms. Unfortunately, there are few tools for the practitioners to use with credibility to date. A preliminary study applies several histogram thresholding methods in detecting appendix [11] but that method is weak when the brightness contrast is not very high and will have potential information loss in edge linking procedure.

Pixel clustering methods [2, 12, 13] are designed to enhance the brightness contrast and form an appendix object from US by using fuzzy binarization [12] or forming object with K-means clustering [13], ART2 neural learning, and fuzzy ART [2] to overcome the subjectivity of US analysis and to extract appendix automatically with high accuracy.

While the recent result [2] demonstrates its superiority in successful extraction rate (high sensitivity) with fuzzy ART over other pixel clustering methods, failed extraction cases suggest that there might be a close relationship between the shape of the appendix in the image and the brightness distribution of the surrounding environments. Thus, in this paper, we propose a more efficient method to extract appendix area correctly by using self-organizing map (SOM) algorithm [14] in the critical pixel clustering process instead of fuzzy ART. SOM shares its structural stability with fuzzy ART as it is relatively not sensitive to the setting of vigilance parameter but it is also a nonlinear, ordered, smooth mapping of high dimensional data onto the regular, low-dimensional array [15]. SOM is an unsupervised learning neural network tool used in many medical image analysis applications successfully [16, 17]. Another advantage of SOM in object clustering over fuzzy ART in this medical application is that SOM can learn more coherent clusters than fuzzy ART in pixel clustering such that it is less sensitive to the shape of the appendix with respect to the brightness distribution of its surroundings. Medical experts suggest that there can be four distinctive shape-brightness types in consideration and those types cover most clinical appendicitis cases in practice. Thus, our method is expected to show more stable performance than previous attempts for various shape patterns of appendices in the US images. Our experiment is designed with respect to such clinical observations in this paper.

**Figure 1:**
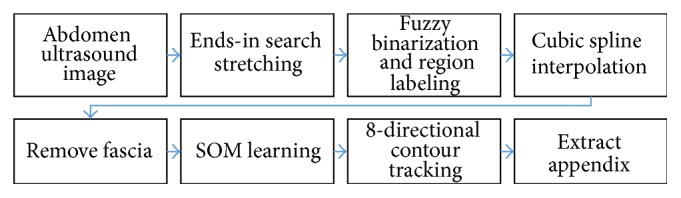
Process of appendix extraction.

Knowing that the appendix is located at the lower organ area below the bottom fascia line, we conduct a series of image processing techniques to find the fascia line correctly and limit the region of interest to form the appendix object accurately.  Figure 1 demonstrates the overall process of our method.

The first step of our automatic extraction methodology is to enhance the brightness contrast by ends-in search stretching [18]. Knowing that the appendix is below fascia area, our method tries to find and remove the fascia area to limit the region of interest. In this part, the bottom fascia lines are carefully treated with cubic spline interpolation [19] such that the lines are correctly connected. The appendix area is then extracted from that image by applying SOM algorithm to form the target object and then the boundary lines are refined by 8-directional contour tracking again.

## 2. Removing Fascia Area after Stretching

**Figure 2:**
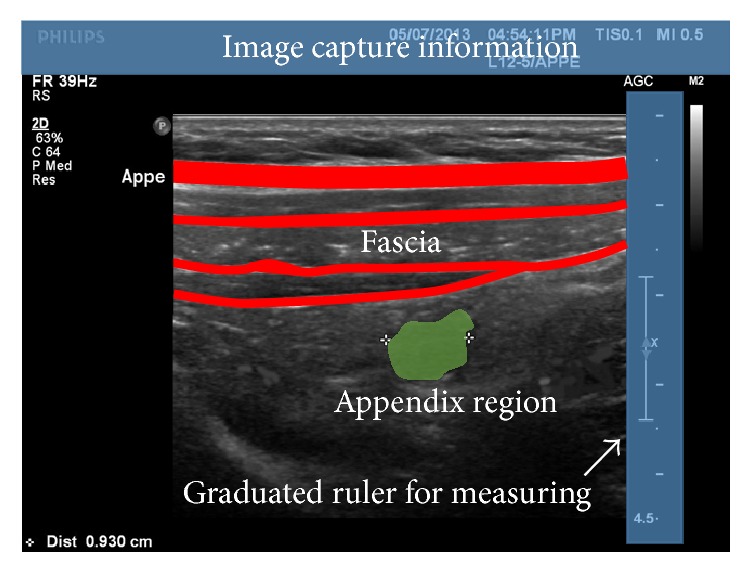
Typical input ultrasound image.

As shown in Figure 2, abdomen ultrasound image consists of image filming information on the above and measurement information on the right and the abdomen image at the center. In the abdomen image, there are fascia area including muscles and appendix area below the fascia. Appendix has the shape of a circle or flat oval.

**Figure 3:**
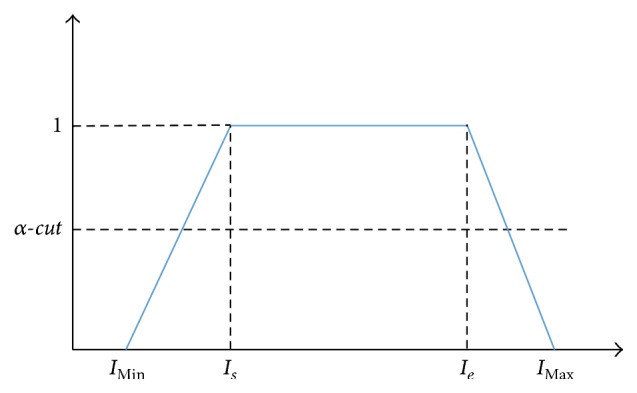
Membership function for fuzzy binarization.

Usually the size of appendix is between 6 mm and 12 mm. After ends-in search stretching for enhancing the brightness contrast and removing unnecessary measuring ruler part from input US as we did in [12], we set up the fuzzy membership function for binarization for our region of interest (ROI). Unlike [12], in this paper, we apply trapezoidal membership function as shown in Figure 3, where *I*_Max_ and *I*_Min_ denote the brightest and the darkest pixel of the ROI, respectively, and let *T* be the average of *I*_Max_ and *I*_Min_. Then, (1) defines the upper bound of trapezoid that has membership degree 1 as interval [*I*_*s*_, *I*_*e*_]

(1) Is=T3,Ie=2Is.

The whole membership degree over interval [*I*_Min_, *I*_*s*_, *I*_*e*_, *I*_Max_] is defined as

(2) $$\begin{array}{c} if\;\;\left(\;I_{Min}<I\leq I_{s}\;\right)\;\;then\;\;\frac{I-I_{s}}{I_{s}-I_{Min}}+1\\ if\;\;\left(\;I_{s}<I\leq I_{e}\;\right)\;\;then\;\;\mu \left(I\right)=1.0\\ if\;\;\left(\;I_{e}<I\leq I_{Max}\;\right)\;\;then\;\;\mu \left(I\right)=\frac{I-I_{e}}{I_{Max}-I_{e}}+1. \end{array}$$

*α-cut* is the median of the interval (0.5) in this paper since there is no specific preference.

**Figure 4:**
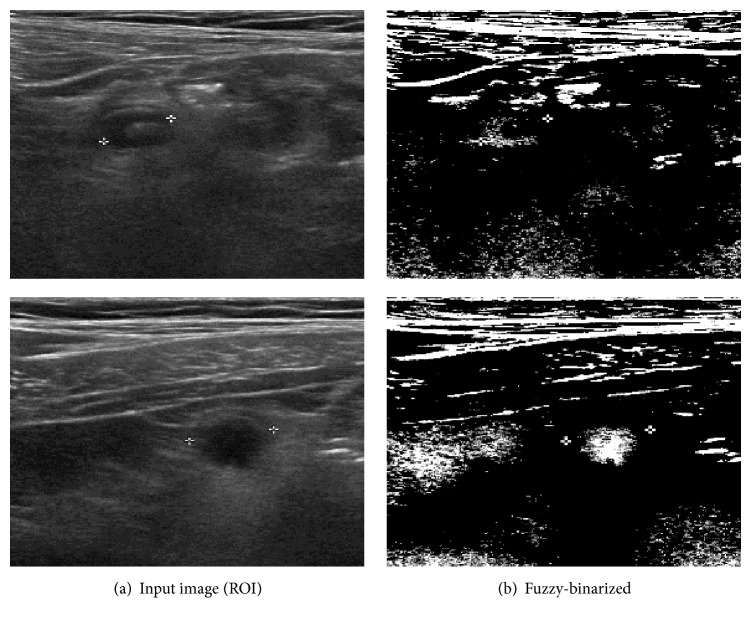
Effect of fuzzy binarization.

The effect of fuzzy binarization is shown in Figure 4.

**Figure 5:**
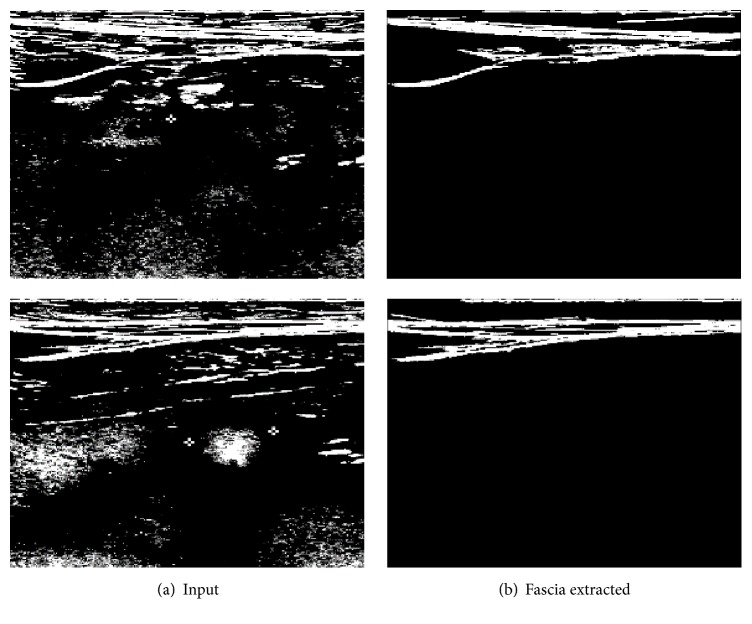
Extracting fascia lines with contour tracking.

From the result of fuzzy binarization, we apply 8-directional contour tracking algorithm [18] to extract fascia boundary lines. Fascia has the shape of a horizontally long thin object; thus we extract such object that is long enough (longer than 1/3 of the width of the ROI in this paper) as fascia.  Figure 5 shows results of 8-directional contour tracking.

**Figure 6:**
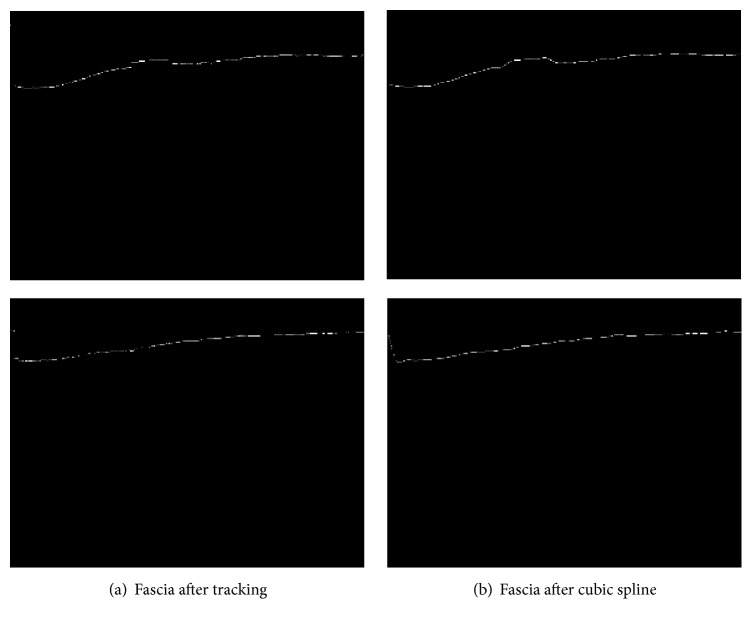
Extraction of fascia boundary line.

Unfortunately, the binarized noiseless image may have disconnected fascia area due to the brightness difference of that area. In order to reconnect them, cubic spline interpolation [19] is applied.  Figure 6 demonstrates the effect of cubic spline interpolation.

Then we remove such fascia area so that the refined ROI is focused on the extraction of appendix.

## 3. Extracting Appendix with Self-Organizing Map

**Figure 7:**
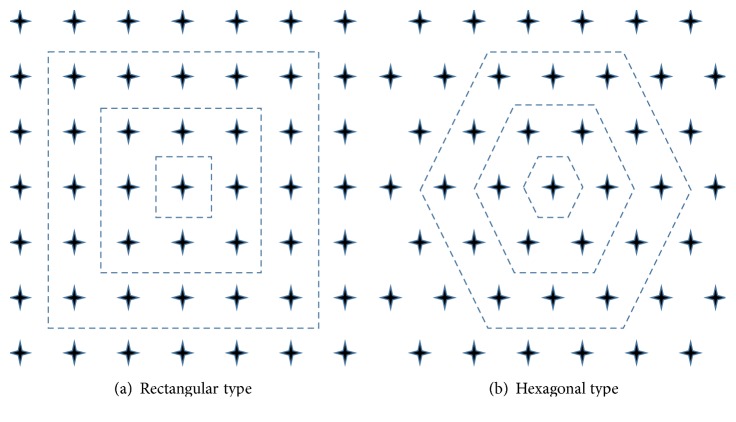
Node arrangement for SOM learning.

SOM algorithm is a time-efficient unsupervised learning algorithm that maps complex multi-dimensional data onto a 2-dimensional space without predefined number of clusters or correlation between data. Like fuzzy ART, SOM also is not sensitive to the setting of vigilance parameter but the arrangement of nodes (neurons) may concern its performance. The usual arrangement of nodes is two-dimensional regular spacing in a hexagonal or rectangular grid as shown in Figure 7 and we choose hexagonal arrangement in this paper.

Since the shape of our target object, appendix, has oval shape, it is more natural to use hexagonal arrangement than rectangular ones. In this quantification process using SOM, we let our SOM learn sufficiently many times in repetition.  Algorithm 1 summarizes our adoption of SOM in this paper.

**Figure 8:**
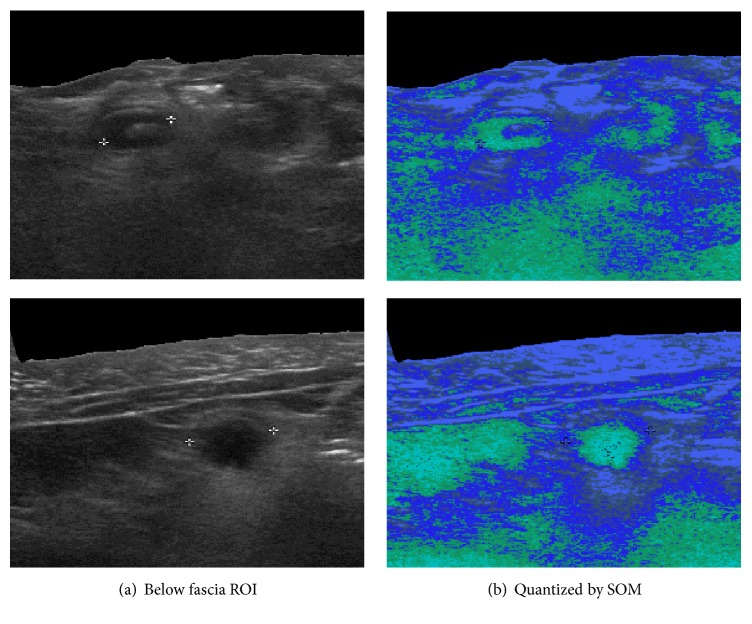
The effect of quantization by SOM.

The effect of quantification by SOM learning is as shown in Figure 8.

**Figure 9:**
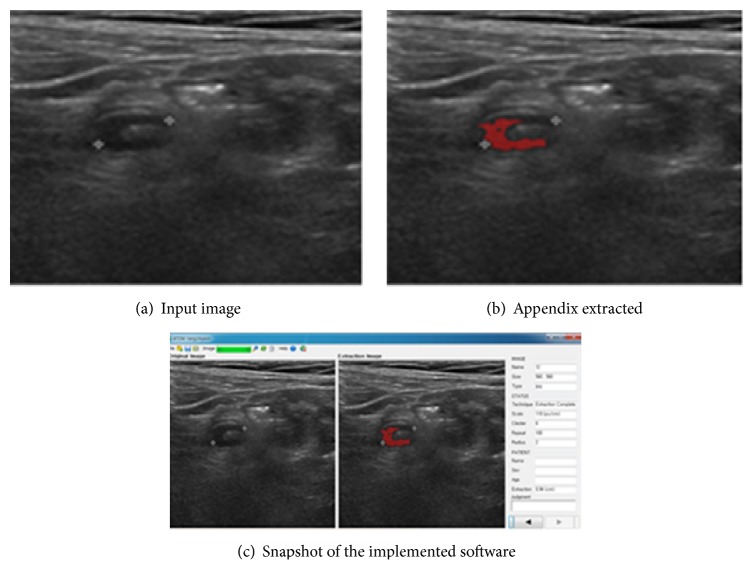
Automatic extraction of appendix: example.

The last part of the appendix extraction process is again 8-directional contour tracking [18].  Figure 9 shows an example of appendix extraction and Figure 9(c) represents a snapshot of implemented software.

## 4. Experiment and Analysis

The system is implemented in Visual Studio 2010 C# with Intel(R) Core(TM) i7-2600 CPU @ 3.40 GHz and 4 GB RAM PC. Sixty images containing appendicitis supplied by Busan Paik Hospital and Busan National University Medical Center, Korea, are used in this experiment. The actual system gives some characteristic features of extracted appendix as shown in Figure 9(c).

**Figure 10:**
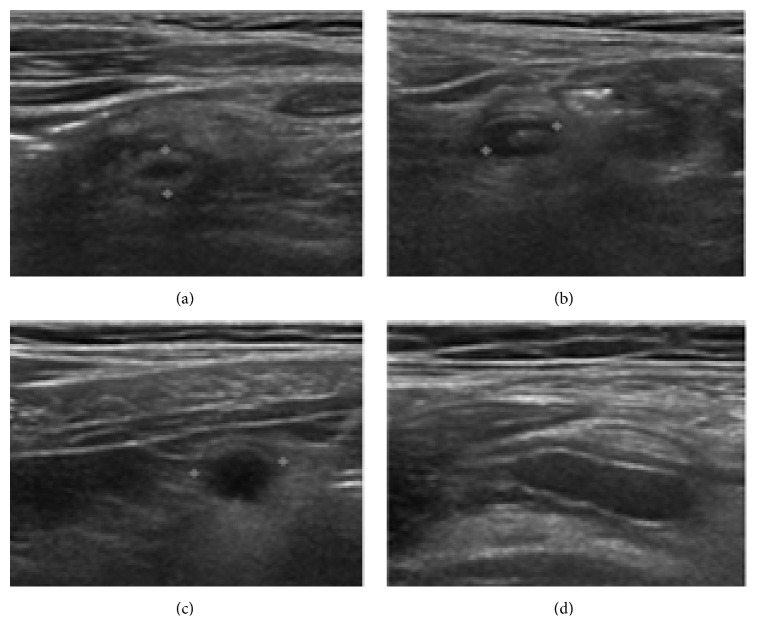
Shape-brightness patterns of appendices.

Medical experts in this field suggest that there are a few shape-brightness pattern types of appendices found in clinical practice. Four patterns shown in Figure 10 are what they observe most frequently. Appendix in type A represents an oval appendix whose surroundings are brighter than the appendix area. Type B represents a hooked shape of appendix. Type C represents also an oval shape appendix but the brightness of the appendix is much lower than the surroundings. Type D, which is the hardest to extract in this experiment, represents a long oval shape appendix whose brightness contrast is very low compared with surroundings. For type D, thus, the shape is more important than the brightness contrast in naked eye inspection but the automatic procedure will suffer the most.

**Figure 11:**
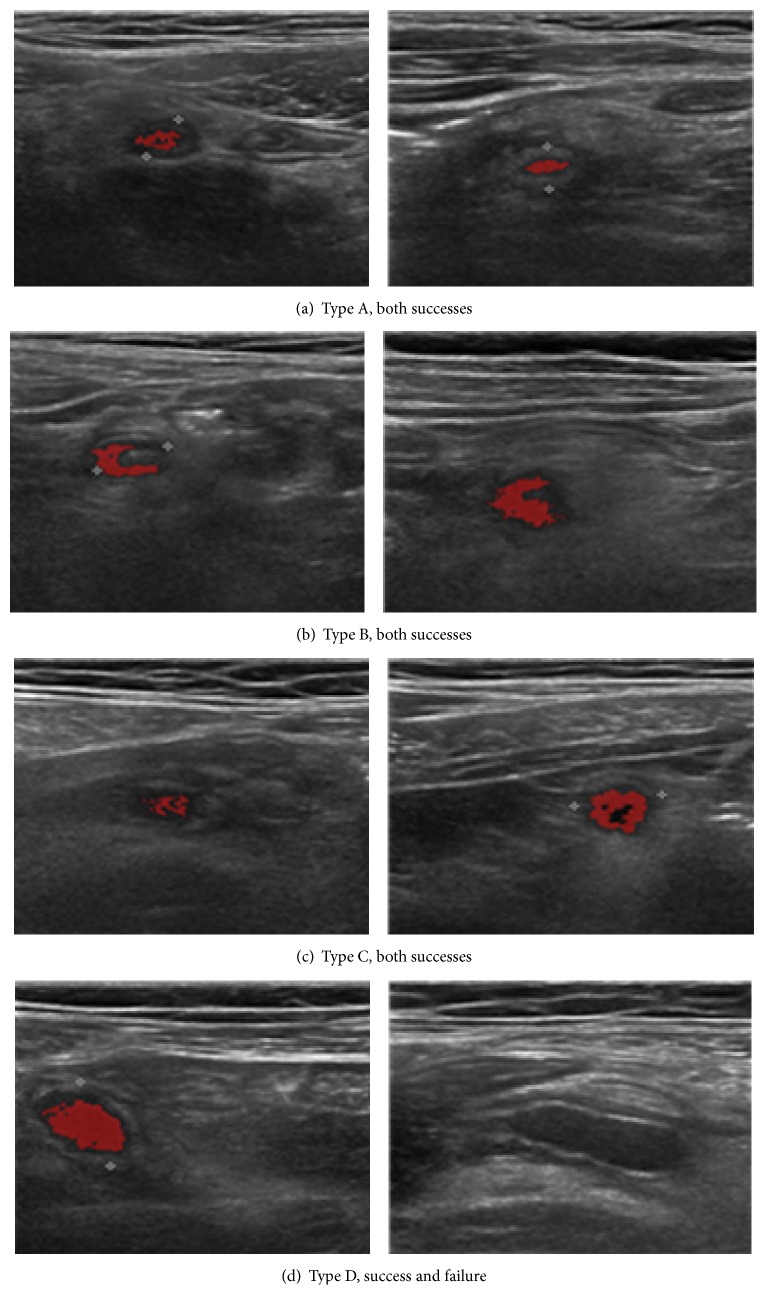
Appendices extractions with respect to the shape patterns.

In experiment, we arrange 15 DICOM US images for each type.  Figure 11 demonstrates the successful and failed extraction cases for each pattern type.

Apparently, type C is the easiest and type D is the hardest for both human expert and the software.  Table 1 summarizes the experimental result of our proposed algorithm comparing with previous fuzzy ART approach [2].

The success and failure decision in this experiment is made by the agreement of multiple medical doctors. In our experiment, there is no true negative input image; thus the specificity is the extraction rate (ext. rate) in Table 1.

**Figure 12:**
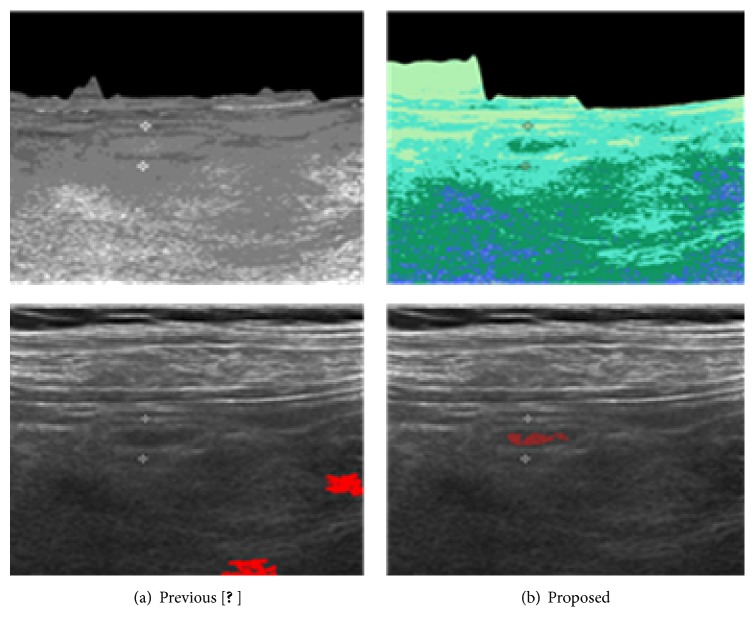
Direct comparison of appendices extraction.

As one can see from Table 1, type C is easy for both algorithms but, in other types, the proposed SOM based learning improves the specificity compared with that of fuzzy ART. The result supports the observation that although fuzzy ART is also a method that is not sensitive to the vigilance parameter settings, low brightness contrast would limit the power of ART learning in clustering phase. In that sense, we confirm that the SOM learning is more stable in clustering.  Figure 12 demonstrates the difference between previous approach [2] and proposed method in quantization (upper image) and the extraction result (lower image).

## 5. Conclusion

In this paper, we propose a method to extract appendix automatically by using a series of image processing algorithms and self-organizing map that learns typical shape patterns of appendix from US. Accurate extraction of appendix area from such appendicitis cases could be critical when the patient is a woman in pregnancy or a young child when that is found as acute appendicitis. Developing such software that extracts target appendix automatically with high accuracy is much needed to avoid operator subjectivity and to sustain high reliability.

The proposed software adopts SOM learning such that it learns the shape patterns and shows stable performance in extracting target appendix accurately in most cases through carefully designed experiment. Extracted appendix results were shown to multiple medical experts and it is regarded as correct extraction when two or more human experts agree that the output from the software is sufficiently accurate. In that regard, our proposed software correctly extracted target appendix of various patterns in 56 cases out of 60 given cases (93.35%) and is completely successful in two out of four types of shape patterns. Such performance is a good improvement from our previous fuzzy ART based methodology. However, the shape type D that has very long oval shape with weak brightness contrast from surroundings still has room for improvement as being only successful in 80% of the given cases.

## Figures and Tables

**Algorithm 1:**
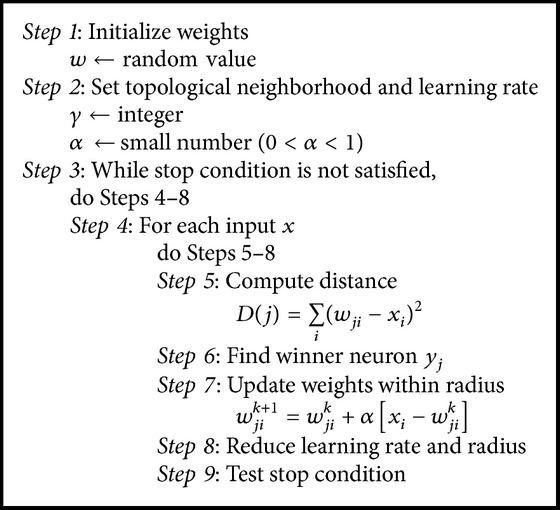
SOM learning algorithm.

**Table 1:** Appendix extraction results.

Type	Previous [2]	Ext. rate	Proposed	Ext. rate
A	13	86.7%	15	100.0%
B	12	80.0%	14	93.3%
C	15	100.0%	15	100.0%
D	9	60.0%	12	80.0%
Total	49	81.7%	56	93.3%
